# Kinetic analysis of endogenous β_2_‐adrenoceptor‐mediated cAMP GloSensor™ responses in HEK293 cells

**DOI:** 10.1111/bph.16008

**Published:** 2023-01-06

**Authors:** Sean A. Cullum, Dmitry B. Veprintsev, Stephen J. Hill

**Affiliations:** ^1^ Division of Physiology, Pharmacology and Neuroscience, School of Life Sciences University of Nottingham Nottingham UK; ^2^ Centre of Membrane Proteins and Receptors University of Birmingham and Nottingham Nottingham UK

**Keywords:** analytical pharmacology, cAMP, signalling kinetics, β_2_‐adrenoceptor

## Abstract

**Background and Aim:**

Standard pharmacological analysis of agonist activity utilises measurements of receptor‐mediated responses at a set time‐point, or at the peak response level, to characterise ligands. However, the occurrence of non‐equilibrium conditions may dramatically impact the properties of the response being measured. Here we have analysed the initial kinetic phases of cAMP responses to β_2_‐adrenoceptor agonists in HEK293 cells expressing the endogenous β_2_‐adrenoceptor at extremely low levels.

**Experimental Approach:**

The kinetics of β_2_‐adrenoceptor agonist‐stimulated cAMP responses were monitored in real‐time, in the presence and absence of antagonists, in HEK293 cells expressing the cAMP GloSensor™ biosensor. Potency (EC_50_) and efficacy (E_max_) values were determined at the peak of the agonist GloSensor™ response and compared to kinetic parameters L_50_ and IR_max_ values derived from initial response rates.

**Key Results:**

The partial agonists salbutamol and salmeterol displayed reduced relative IR_max_ values (with respect to isoprenaline) when compared with their E_max_ values. Except for the fast dissociating bisoprolol, preincubation with β_2_‐adrenoceptor antagonists produced a large reduction in the isoprenaline peak response due to a state of hemi‐equilibrium in this low receptor reserve system. This effect was exacerbated when IR_max_ parameters were measured. Furthermore, bisoprolol produced a large reduction in isoprenaline IR_max_ consistent with its short residence time.

**Conclusions and Implications:**

Kinetic analysis of real‐time signalling data can provide valuable insights into the hemi‐equilibria that can occur in low receptor reserve systems with agonist–antagonist interactions, due to incomplete dissociation of antagonist whilst the peak agonist response is developing.

## Graphical abstract

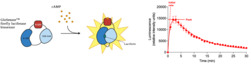

**Abbreviations**
DMEM: Dulbecco's Modified Eagles Medium
E_max_
: maximal response
HBSS: HEPES buffered saline solution
HEK293 cells: Human Embryonic Kidney 293 cells
IBMX: 3‐isobutyl‐1‐methylxanthine
ICI‐118551: (±)‐1‐[2,3‐(dihydro‐7‐methyl‐1*H*‐inden‐4‐yl)oxy]‐3‐[(1‐methylethyl)amino]‐2‐butanol hydrochloride
IR_max_
: maximal initial rate
L_50_
: half‐maximal initial rate concentration
RIIβB: cAMP‐binding domain B from PKA regulatory subunit

**What is already known**

- Intracellular β_2_‐adrenoceptor‐mediated cAMP responses are transient in nature.
- Peak amplitudes of responses are often measured which assume equilibrium and lack of regulatory mechanisms.

**What this study adds**

- Kinetic analysis of responses has enabled comparisons of peak and initial rates of cAMP production.
- This provides valuable insights into the hemi‐equilibria that can occur in low receptor reserve systems.

**Clinical significance**

- β_2_‐adrenoceptor agonists and antagonists are effective treatments for respiratory and cardiovascular diseases, respectively.
- The kinetic properties of antagonists influence the antagonism produced in cells with low receptor expression.

## 1 INTRODUCTION

The β_2_‐adrenoceptor is a member of the large G protein‐coupled receptor (GPCR) family of membrane proteins (Fredriksson et al., 2003; Lagerström & Schiöth, 2008). GPCRs represent the greatest target for therapeutics, accounting for approximately one third of all current FDA‐approved drugs (Santos et al., 2017; Sriram & Insel, 2018). The β_2_‐adrenoceptor is expressed predominantly in airway and vascular smooth muscle cells, as well as in the heart and inflammatory cells (Billington et al., 2013; Feldman & Gros, 1998; Pérez‐Schindler et al., 2013; Tanaka et al., 2005), and has been targeted successfully by β‐agonists for the treatment of asthma and other pulmonary diseases (Billington et al., 2013; Bosmann et al., 2012; Minneman et al., 1981). The β_2_‐adrenoceptor signals primarily through its coupling to the heterotrimeric G_s_ protein, which activates adenylate cyclase to increase production of the intracellular second messenger cAMP (Neves et al., 2002; Rasmussen et al., 2011; Tanaka et al., 2005), although it also couples to β‐arrestin which causes receptor desensitisation, internalisation and is involved in alternate signalling pathways (Shenoy & Lefkowitz, 2011; Shukla et al., 2014).

It has been common practice in pharmacology to utilise measurements of receptor‐mediated responses at different ligand concentrations at a set time‐point, or at the peak response level, to produce concentration‐response curves from which ligand parameters such as potency (EC_50_) and maximal response (E_max_) can be calculated (Black & Leff, 1983; Finlay et al., 2020; Hoare et al., 2022; Stephenson, 1956; Zhu et al., 2019). This analysis has allowed the relative activities of different ligands to be compared and has uncovered mechanistic insights into ligand‐receptor interactions, imperative for the development of improved therapeutics (Kenakin, 2019). However, this classic pharmacological analysis assumes equilibrium conditions have been reached in the system, which is not always the case, and cannot distinguish the generation of the signal by the agonist‐occupied receptor from the counteractive regulatory mechanisms which diminish the signal such as receptor desensitisation and signal degradation (e.g., breakdown of second messenger molecules) (Hoare et al., 2020, 2022; Moore, Milano, & Benovic, 2007; Zhu et al., 2019). Thus, peak response measurements taken from non‐equilibrium conditions, or measurements taken at distinct time‐points which are differentially affected by regulatory mechanisms, may distort calculated parameters such as potency and efficacy (Bdioui et al., 2018; Hoare et al., 2022; Klein Herenbrink et al., 2016; Zhu et al., 2019).

The recent development of new and improved biosensor technologies has enabled the continuous measurement of GPCR signals, thus allowing quantification of the entire time‐course of the response (Goulding et al., 2018; Greenwald et al., 2018; Lohse et al., 2008, 2012; Wright & Bouvier, 2021). The derivation of equations to fit these time‐course data has made it possible to estimate kinetic signalling parameters such as kinetic potency (L_50_) and maximal initial rate (IR_max_), which is related to efficacy (Hoare et al., 2020, 2022). This kinetic analysis could uncover new information about the pharmacological and kinetic properties of ligands, which may ultimately allow for more accurate characterisation of ligand‐receptor interactions.

In this study, we have monitored β_2_‐adrenoceptor‐mediated cAMP responses in HEK293 cells using the cAMP GloSensor™ biosensor (Binkowski et al., 2011; Fan et al., 2008). This biosensor consists of a firefly luciferase enzyme genetically fused to the cAMP‐binding domain of a protein kinase A (PKA) regulatory subunit (RIIβB) (Binkowski et al., 2011; Fan et al., 2008). Upon cAMP binding, the luciferase undergoes a conformational change which in the presence of the luciferase substrate results in an increase in luminescence emission (Binkowski et al., 2011; Fan et al., 2008). Here, we have monitored the kinetics of agonist‐mediated β_2_‐adrenoceptor responses using the approach of Hoare et al. (2020) and compared the parameters determined with the equivalent classic pharmacological parameters (EC_50_, E_max_) determined from measurement of peak responses that assume equilibrium conditions. HEK293 cells endogenously express the β_2_‐adrenoceptor at extremely low levels (Friedman et al., 2002; Goulding, Kondrashov, et al., 2021, Goulding, Mistry, et al., 2021) and this coupled with real‐time monitoring of cAMP generation allowed us to compare the impact of signalling kinetics on the pharmacological parameter estimates of the full agonists isoprenaline and formoterol and the partial agonists salbutamol and salmeterol. In addition, the effect of competing β_2_‐adrenoceptor antagonists of differing dissociation rates on isoprenaline‐response kinetic parameters have been investigated under hemi‐equilibrium conditions.

## 2 METHODS

### 2.1 Chemicals and reagents

The cAMP GloSensor™ Human Embryonic Kidney 293 (HEK293G) cell line and GloSensor™ cAMP reagent were purchased from Promega (Madison, WI, USA). Isoprenaline hydrochloride, salmeterol, ICI‐118551, propranolol, bisoprolol, 3‐isobutyl‐1‐methylxanthine (IBMX), rolipram, Dulbecco's Modified Eagle Medium (DMEM), L‐glutamine, phosphate buffered saline (PBS), trypsin–EDTA, foetal calf serum (FCS) and poly‐D‐lysine were all obtained from Sigma‐Aldrich (St. Louis, MO, USA). Forskolin, formoterol and salbutamol hemisulfate were obtained from Tocris Bioscience (Bristol, UK). Carvedilol was obtained from ACROS Organics (Geel, Belgium). Any other chemicals used were from Sigma‐Aldrich (St. Louis, MO, USA).

### 2.2 Cell culture

HEK293 cells stably expressing the cAMP GloSensor™ (20F) biosensor obtained from Promega (Madison, WI, USA) were termed HEK293G cells. HEK293G cells were maintained in DMEM supplemented with 2 mM L‐glutamine and 10% FCS at 37°C and 5% CO_2_. Cells were grown in sterile conditions in uncoated T75 tissue culture flasks. Once confluent, cells were washed with PBS and dislodged from the flask surface by incubation with 1× trypsin–EDTA in PBS, then pelleted by centrifugation for 4 min at 1000× *g* followed by resuspension in DMEM supplemented with 2 mM L‐glutamine and 10% FCS. Cells were then either split to a lower cell density and returned to a new T75 flask for continuation of the cell line or seeded at 30,000 cells per well into white walled, clear bottomed 96‐well plates (pre‐treated with 10 μg·ml^−1^ poly‐D‐lysine for improved cell adhesion to the well surface) with 100 μl media per well. Cell densities were calculated using a haemocytometer. The seeded plates were then incubated at 37°C and 5% CO_2_ for 24 h prior to assay.

### 2.3 cAMP GloSensor™ luminescence assay

The cAMP GloSensor™ luminescence assay was performed according to the manufacturer's instructions (Promega, Madison, WI, USA). Briefly, after 24 h incubation at 37°C and 5% CO_2_ after cell plating, media was aspirated from each well of the 96‐well plate. Cells were incubated in 50 μl HEPES buffered saline solution (HBSS; 2 mM sodium pyruvate, 145 mM NaCl, 10 mM D‐glucose, 5 mM KCl, 1 mM MgSO_4_.7H_2_O, 10 mM HEPES, 1.3 mM CaCl_2_, 1.5 mM NaHCO_3_ in double‐distilled water, pH 7.45) containing 3% GloSensor™ cAMP reagent at 37°C and 5% CO_2_ for 2 h. A white seal was placed on the back of the plate before reading. For agonist studies, luminescence was measured immediately after addition of a further 50 μl HBSS containing agonist (2× final concentration) or HBSS (vehicle control). Luminescence was measured continuously over 60 min, reading each well once every minute, by a PHERAstar FSX microplate reader (BMG Labtech, Offenburg, Germany). Increases in luminescence are indicative of intracellular cAMP accumulation, thus the temporal changes in relative cytosolic cAMP concentration were measured upon agonist or vehicle addition. Baseline luminescence was measured in each well prior to addition. For phosphodiesterase (PDE) inhibitor or agonist versus antagonist/inverse agonist studies, the same process was performed with the additional preincubation of 5 μl HBSS containing PDE inhibitor, antagonist/inverse agonist (20× final concentration) or vehicle, 30 min prior to application of agonist (2× final concentration) or vehicle. All conditions were performed in three to six replicates within each plate.

### 2.4 Data analysis and statistics

Data were analysed and presented using GraphPad Prism 8 software (San Diego, CA, USA). Results are generally expressed as mean ± standard error of mean (SEM) from five separate experiments, unless otherwise stated. The number of independent experiments ‘n’ is stated throughout and statistical analysis was only performed on data where n = 5. In addition, any outliers were included within the data analysis and presentation throughout the study. Peak responses were determined as the maximal signal in each trace. Parallel measurements were made at each time‐point following addition of HBSS in place of agonist under the same experimental conditions. These values were subtracted from the equivalent agonist‐induced data at each time‐point to provide a baseline‐corrected time‐course. Statistical analyses were also performed using GraphPad Prism 8 software (San Diego, CA, USA). Statistical significance of data was tested using either unpaired, two‐tailed *t*‐test or one‐way ANOVA and Tukey's multiple comparisons test. Post‐hoc tests were run only if F achieved P<0.05 and there was no significant variance inhomogeneity. Throughout the study, *P* < 0.05 was used as the level for significance. The data and statistical analysis comply with the recommendations of the *British Journal of Pharmacology* on experimental design and analysis in pharmacology (Curtis et al., 2022).

The Hill equation, shown in Equation (1), was used to fit concentration‐response data to a standard sigmoidal curve, where ‘*E*’ represents the magnitude of response, ‘*E*_*max*_’ represents the maximal response magnitude, ‘[*A*]’ is the ligand concentration, ‘*EC*_*50*_’ is the half‐maximal response concentration and ‘*n*’ is the Hill coefficient.

(1) $$\frac{E}{E_{\max}}=\frac{{\left[A\right]}^{n}}{{\mathrm{EC}}_{50}^{n}+{\left[A\right]}^{n}}$$

The ‘rise‐and‐fall‐to‐baseline time‐course’ equation, shown in Equation (2), was used to fit time‐course data to a kinetic curve, according to Hoare et al. (2020), where ‘*IR*’ is a fitting constant (in units of y‐units.t^−1^), which is equal to the initial rate of signalling, the initial linear phase of signal generation upon ligand addition. ‘*k*_1_’ and ‘*k*_2_’ are two regulatory rate constants (in units of t^−1^) which are responsible for attenuating the initial rate of response (e.g., due to desensitisation) and the decay of the cAMP response (e.g., due to phosphodiesterase activity), which cause the signal to peak and then decline back towards baseline (Hoare et al., 2020). Equation (2) was provided as a plug‐in which was downloaded into GraphPad Prism 8 software (San Diego, CA, USA; Hoare et al., 2022). *k*_1_ was assumed to be the larger of the two rate constant values and this was handled by constraining *k*_1_ to be greater than *k*_2_. In all cases, rate constant values were constrained to be greater than zero.

(2) $$y=\frac{\mathrm{IR}}{k_{1}-k_{2}}\;\left(e^{-k_{2}t}-e^{-k_{1}t}\right)+\text{Baseline}$$

Concentration‐response data for the initial rates were fit to a variable slope Hill equation, displayed in Equation (3), where ‘*IR*’ represents the initial rate of signalling, ‘*IR*_*max*_’ is the maximal initial rate response, ‘[*A*]’ is the ligand concentration, ‘*L*_*50*_ *’* is the half maximal initial rate concentration and ‘*n*’ is the Hill coefficient.

(3) $$\frac{\mathrm{IR}}{{\mathrm{IR}}_{\max}}=\frac{{\left[A\right]}^{n}}{L_{50}^{n}+{\left[A\right]}^{n}}$$

### 2.5 Nomenclature of targets and ligands

Key protein targets and ligands in this article are hyperlinked to corresponding entries in http://www.guidetopharmacology.org, and are permanently archived in the Concise Guide to PHARMACOLOGY 2021/22 (Alexander, Christopoulos, et al., 2021; Alexander, Fabbro, et al., 2021).

## 3 RESULTS

### 3.1 Characterisation of peak β_2_‐adrenoceptor‐mediated cAMP responses

**FIGURE 1:**
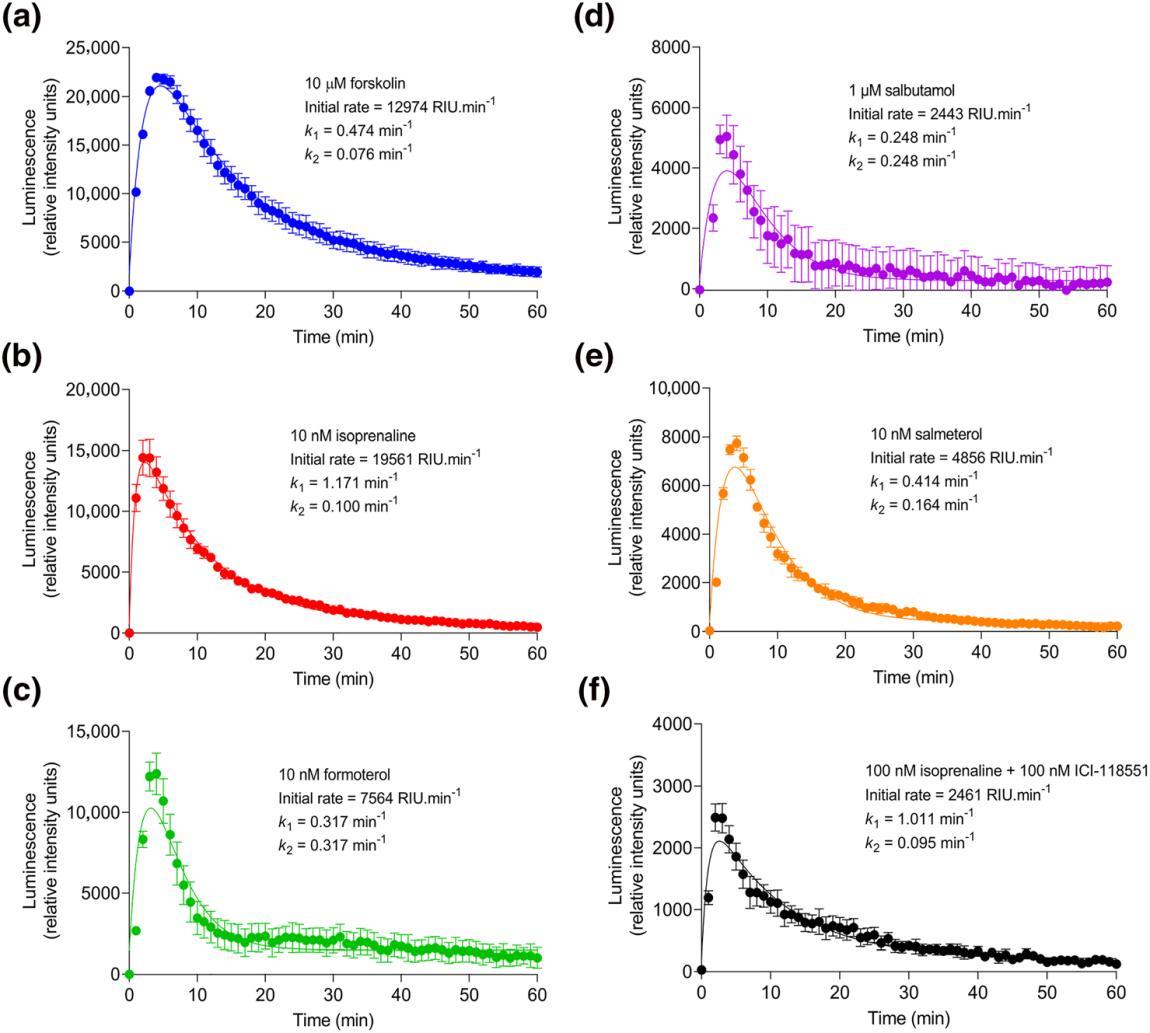
GloSensor™ luminescence stimulated by forskolin‐, isoprenaline‐ formoterol‐, salbutamol‐ and salmeterol‐mediated cAMP production. Representative GloSensor™ luminescence time‐course data in one experiment over 60 min following application of (a) forskolin (10 μM), (b) isoprenaline (10 nM), (c) formoterol (10 nM), (d) salbutamol (1 μM), (e) salmeterol (10 nM) and (f) isoprenaline (100 nM) in the presence of preincubated ICI‐118551 (100 nM) to HEK293G cells, fitted with time‐course curve fitting according to Hoare et al. (2020). Derived kinetic parameters (initial rate, *k*_1_ and *k*_2_ values) are displayed. Data are mean ± standard error of mean (SEM) of triplicate measurements, expressed as relative intensity units (RIU) of luminescence. Similar data were obtained in five independent experiments.

**FIGURE 2:**
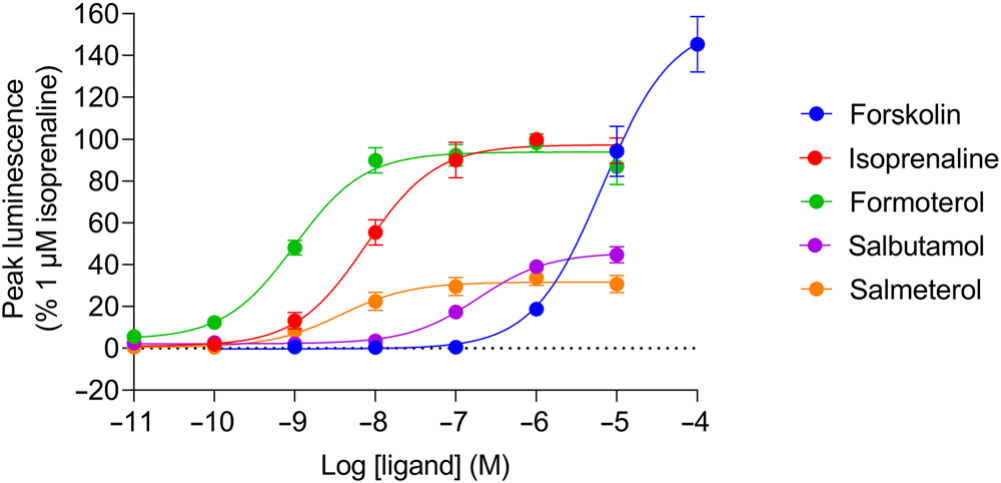
GloSensor™ luminescence stimulated by forskolin‐, isoprenaline‐ formoterol‐, salbutamol‐ and salmeterol‐mediated cAMP production. Concentration‐response curves for mean peak responses to forskolin (100 pM–100 μM), isoprenaline, formoterol, salbutamol and salmeterol (all 10 pM–10 μM) expressed as a percentage of the 1 μM isoprenaline response obtained in each individual experiment. Data points represent mean ± SEM from five independent experiments (n = 5).

Figure 1 shows representative transient changes in GloSensor™ luminescence measured over 60 min upon application of forskolin, isoprenaline (both in the presence and absence of ICI‐118551), formoterol, salbutamol and salmeterol to HEK293G cells, which endogenously express the β_2_‐adrenoceptor at extremely low levels (Friedman et al., 2002; Goulding, Kondrashov, et al., 2021, Goulding, Mistry, et al., 2021). These time‐course data are indicative of changes in cytosolic cAMP concentrations. Both direct activation of adenylate cyclase by forskolin (Seamon & Daly, 1981) and indirect activation by β_2_‐adrenoceptor ligands stimulated a rapid increase in luminescence to a peak response followed by a decline of the signal back to the baseline. The relative peak luminescence produced by increasing concentrations of the ligands were normalised against 1 μM isoprenaline and fitted to a standard sigmoidal curve using the Hill equation (Equation 1), displayed in Figure 2. Each ligand stimulated a concentration‐dependent peak response. The calculated potencies (log EC_50_) and relative maximal responses (E_max_) for each of the β_2_‐adrenoceptor ligands are displayed in Table 1; 100 μM forskolin produced the largest peak response. The β_2_‐adrenoceptor partial agonists salbutamol and salmeterol produced considerably reduced maximal responses compared with isoprenaline and formoterol (*P* < 0.05).

**TABLE 1:** Agonist log E_max_, IR_max_, log EC_50_ and log L_50_ values ± SEM determined for isoprenaline, formoterol, salbutamol and salmeterol from concentration‐response curves obtained by cAMP GloSensor™ in HEK293G cells from five independent experiments (n = 5)

Ligand	E_max_ (% 1 μM isoprenaline)	IR_max_ (% 1 μM isoprenaline)	Log EC_50_ (M)	Log L_50_ (M)
Isoprenaline	100	100	−8.01 ± 0.12	−8.13 ± 0.12
Formoterol	98.38 ± 4.31	83.36 ± 7.62	−9.00 ± 0.04	−8.80 ± 0.07
Salbutamol	44.74 ± 3.80	30.34 ± 2.75	−6.73 ± 0.01	−6.68 ± 0.14
Salmeterol	33.73 ± 3.60	22.41 ± 2.16	−8.39 ± 0.12	−8.08 ± 0.11

### 3.2 Kinetic analysis of initial rates of β_2_‐adrenoceptor mediated cAMP responses

**FIGURE 3:**
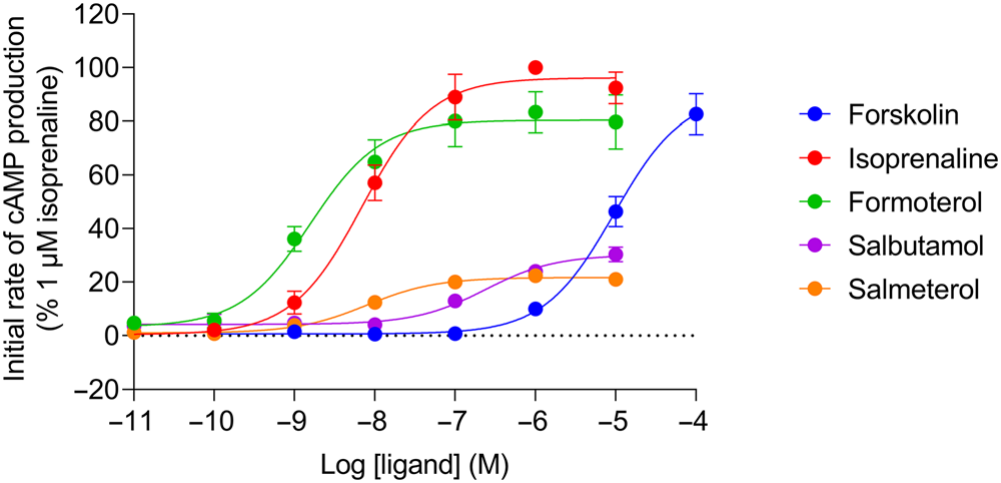
GloSensor™ luminescence stimulated by forskolin‐, isoprenaline‐, formoterol‐, salbutamol‐ and salmeterol‐mediated c AMP production. Concentration‐response curves of initial response rates for forskolin (100 pM–100 μM), isoprenaline, formoterol, salbutamol and salmeterol (all 10 pM–10 μM) expressed as a percentage of the 1 μM isoprenaline response obtained in each individual experiment. Data points represent mean ± SEM from five independent experiments (n = 5).

**FIGURE 4:**
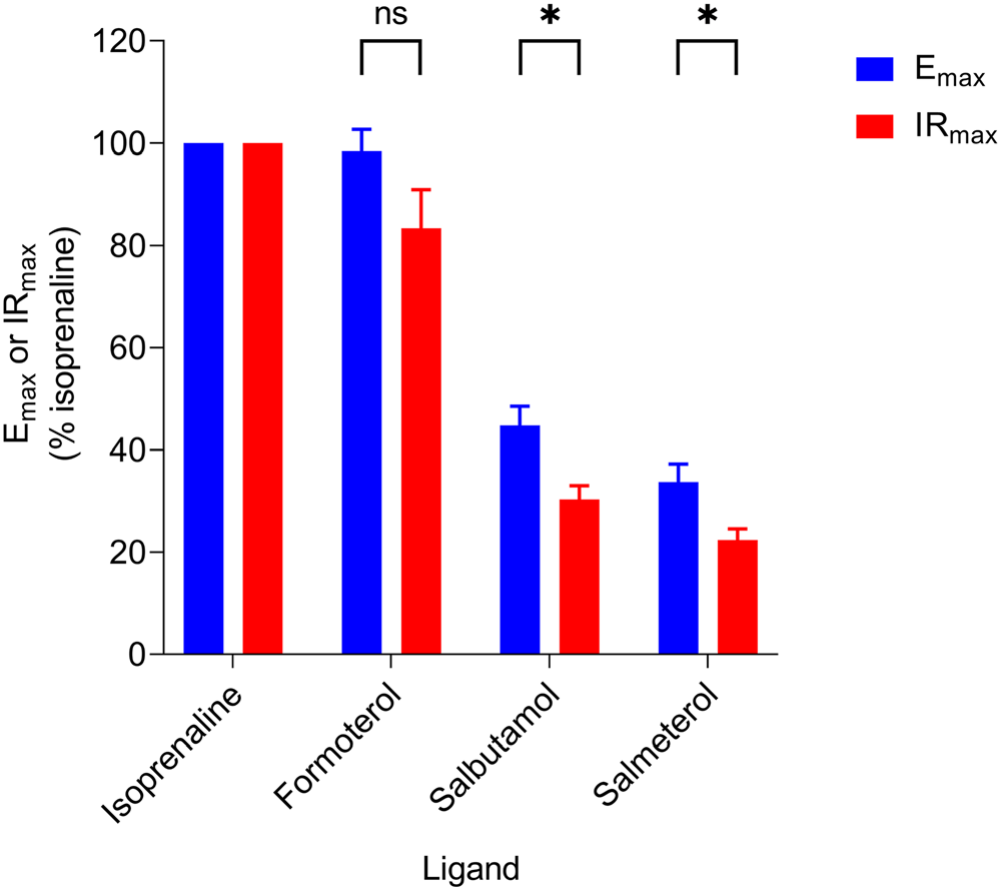
Comparisons of mean E_max_ and IR_max_ values for isoprenaline, formoterol, salbutamol and salmeterol, relative to isoprenaline. Data points represent mean ± SEM from five independent experiments (n = 5). Significant differences are indicated, determined by an unpaired *t*‐test. *P* < 0.05 was used as the level for significance (*P* > 0.05 = no significance [ns], *P < 0.05).

Agonist‐induced cAMP signals were also analysed kinetically to determine initial rates of signal generation (Figure 1). Similar to peak response measurements (Figure 2), the initial rate values for each ligand were concentration‐dependent and could also be fitted to a sigmoidal curve using the modified Hill equation (Equation 3), shown in Figure 3. All conditions were normalised to 1 μM isoprenaline for comparison with peak response data. The kinetic potencies (log L_50_) and maximal initial rates (IR_max_) of the ligands were determined and are displayed in Table 1 for comparison with the log EC_50_ and E_max_ values calculated from Figure 2. The maximal initial rates of salbutamol and salmeterol were much reduced compared with isoprenaline and formoterol (*P* < 0.05) consistent with their partial agonism. In Figure 4, the E_max_ and IR_max_ values are directly compared for each β_2_‐adrenoceptor ligand, normalised against the reference ligand isoprenaline. Both salbutamol and salmeterol showed significantly reduced IR_max_ values compared to their E_max_ values (*P* < 0.05), relative to isoprenaline, whereas formoterol showed no significant difference.

### 3.3 Effect of the phosphodiesterase (PDE) inhibitors IBMX and rolipram on the kinetic profiles of the response to 1 μM isoprenaline

**FIGURE 5:**
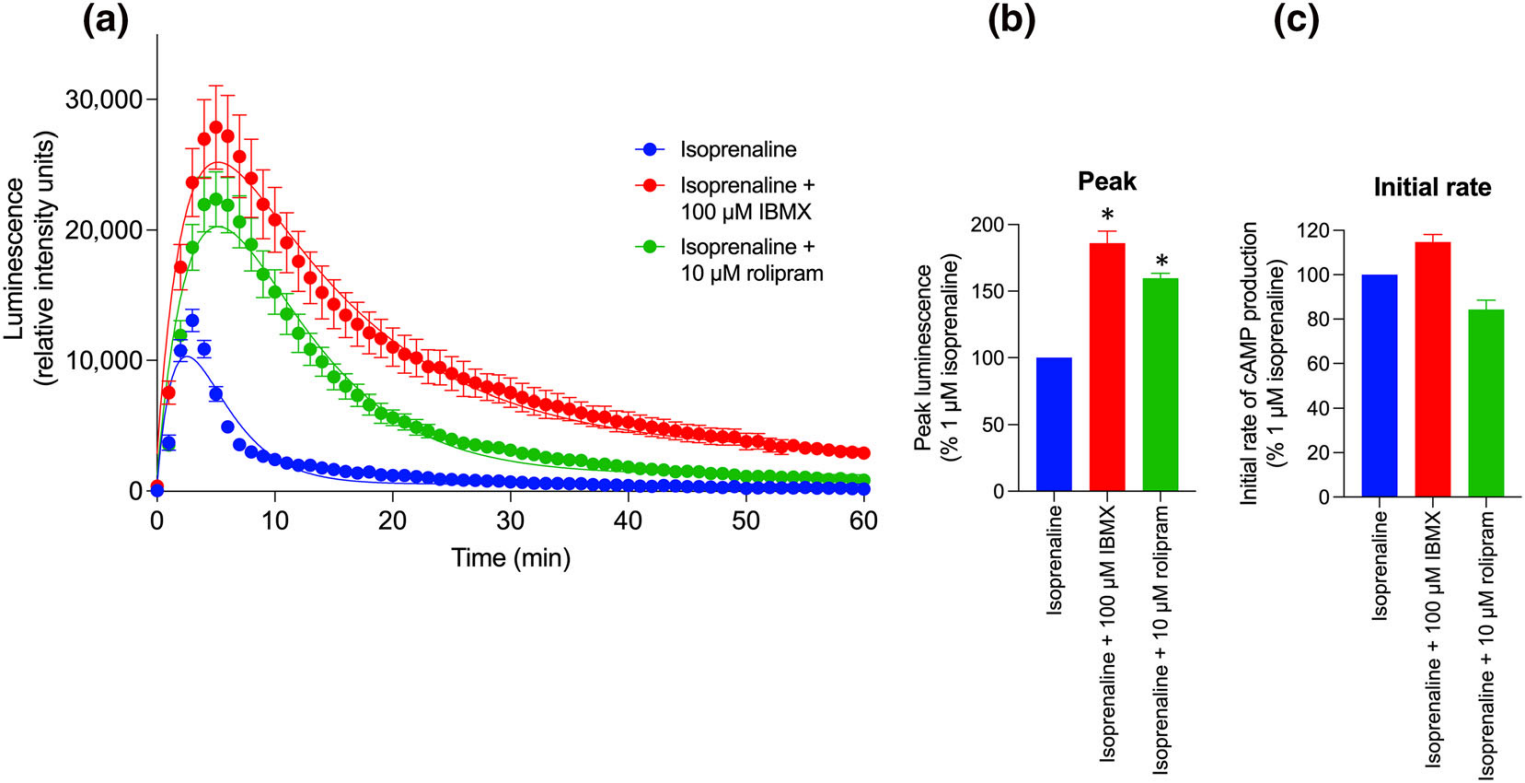
GloSensor™ luminescence stimulated by isoprenaline in the presence and absence of 100 μM IBMX or 10 μM rolipram. (a) Representative GloSensor™ luminescence time‐course data in one experiment over 60 min following application of isoprenaline (1 μM) in the presence or absence of preincubated IBMX (100 μM) or rolipram (10 μM) to HEK293G cells, fitted with time‐course curve fitting according to Hoare et al. (2020). Data are mean ± standard error of mean (SEM) of six replicate measurements, expressed as relative intensity units (RIU) of luminescence. Similar data were obtained in five separate experiments. (b, c) Bar charts displaying (b) mean peak responses and (c) mean initial rates of signal generation for isoprenaline (1 μM) in the presence or absence of preincubated IBMX (100 μM) or rolipram (10 μM) obtained in five separate experiments expressed as a percentage of the 1 μM isoprenaline response obtained in each individual experiment. Data points represent mean ± SEM from five independent experiments (n = 5). Significant difference in isoprenaline peak responses and initial rates of cAMP were determined by a one‐way ANOVA with Tukey's multiple comparisons test. *P* < 0.05 was used as the level for significance (*P < 0.05).

The agonist‐induced cAMP signals generated in this study were all characterised by an initial rise followed by a fall to baseline that was fitted to an equation that described an initial rate of cAMP formation that was subsequently modified by two rate constants (*k*_1_ and *k*_2_) that describe a decline in the cAMP response (e.g., due to phosphodiesterase activity or receptor desensitisation), which cause the signal to decline back towards baseline (Hoare et al., 2020). We have not assigned *k*_1_ and *k*_2_ to particular activities and thus they represent operational rate constants that describe processes that attenuate cAMP generation. To shed some light on the mechanisms involved we have investigated the effect of two PDE inhibitors on the response to 1 μM isoprenaline (Figure 5). Both the selective PDE4 inhibitor rolipram and the general inhibitor IBMX caused a large and significant (*P* < 0.05) increase in the measured peak response to isoprenaline alongside a prolongation of the fall towards baseline (Figure 5a,b; Table 2). In marked contrast, the initial rate of cAMP production was not significantly different in the presence or absence of PDE inhibitors (as would be expected) (Figure 5c). Kinetic analysis of these data also picked up significant changes in one of the rate constants describing the reduction in cAMP levels (Table 2). Thus, rolipram significantly changed *k*_1_, whilst IMBX had a significant effect on *k*_2_ (Table 2). For this reason, we have concentrated in this study on the impact of different treatments on the initial rate of response and treated *k*_1_ and *k*_2_ as operational rate constants that help us define the initial rate of response.

**TABLE 2:** Isoprenaline peak response, initial rate, *k*_1_ and *k*_2_ values ± SEM in the presence and absence of increasing concentrations of the phosphodiesterase inhibitor IBMX obtained by cAMP GloSensor™ in HEK293G cells from five independent experiments (n = 5)

Condition	Peak response (% 1 μM isoprenaline)	Initial rate (% 1 μM isoprenaline)	*k*_1_ (min^−1^)	*k*_2_ (min^−1^)
Isoprenaline	100	100	0.50 ± 0.20	0.38 ± 0.04
Isoprenaline + 10 μM Rolipram	159.7 ± 3.7^*^	84.4 ± 4.2	0.23 ± 0.05^*^	0.17 ± 0.02
Isoprenaline + 100 μM IBMX	186.0 ± 9.0^*^	114.8 ± 3.4	0.44 ± 0.10	0.09 ± 0.01^*^

*Note*: Significant difference in isoprenaline peak response, initial rate, *k*_1_ and *k*_2_ values to those seen in the absence of antagonist are indicated, determined by a one‐way ANOVA with Tukey's multiple comparisons test. *P* < 0.05 was used as the level for significance.

^*^ *P* < 0.05.

### 3.4 Determining the effect of competing antagonists on isoprenaline‐stimulated β_2_‐adrenoceptor activity in a low receptor reserve system

**FIGURE 6:**
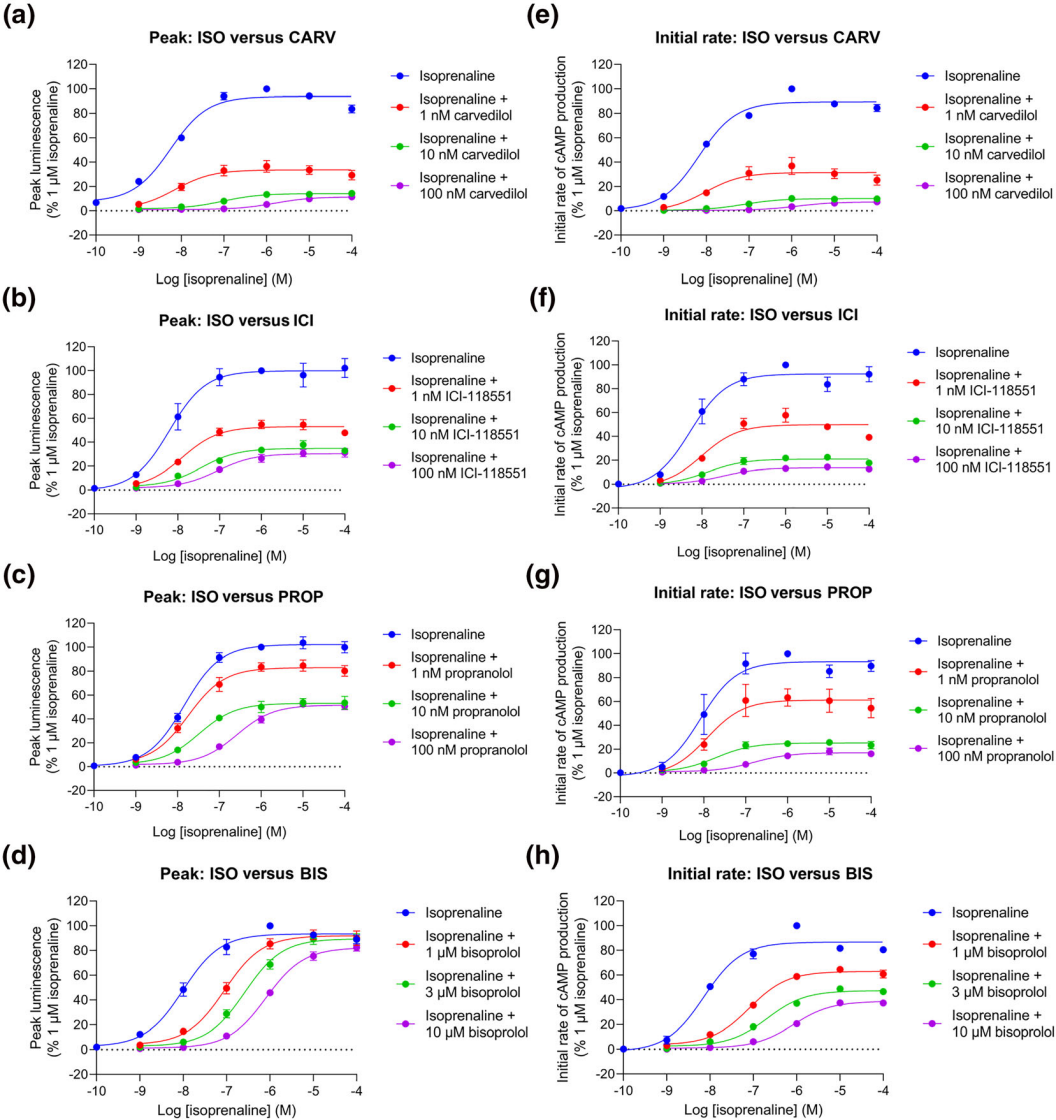
GloSensor™ luminescence stimulated by isoprenaline in the presence and absence of increasing concentrations of various β_2_‐adrenoceptor antagonists/inverse agonists. (a–d) Mean peak concentration‐response curves and (e–h) mean initial rates of signal generation concentration‐response curves for isoprenaline (ISO)(100 pM–100 μM) versus (a, e) carvedilol (CARV); (b, f) ICI‐118551 (ICI); (c, g) propranolol (PROP)(all 1–100 nM) and (d, h) bisoprolol (BIS)(1–10 μM) as a percentage of 1 μM isoprenaline response. Data points represent mean ± SEM from five independent experiments (n = 5).

**FIGURE 7:**
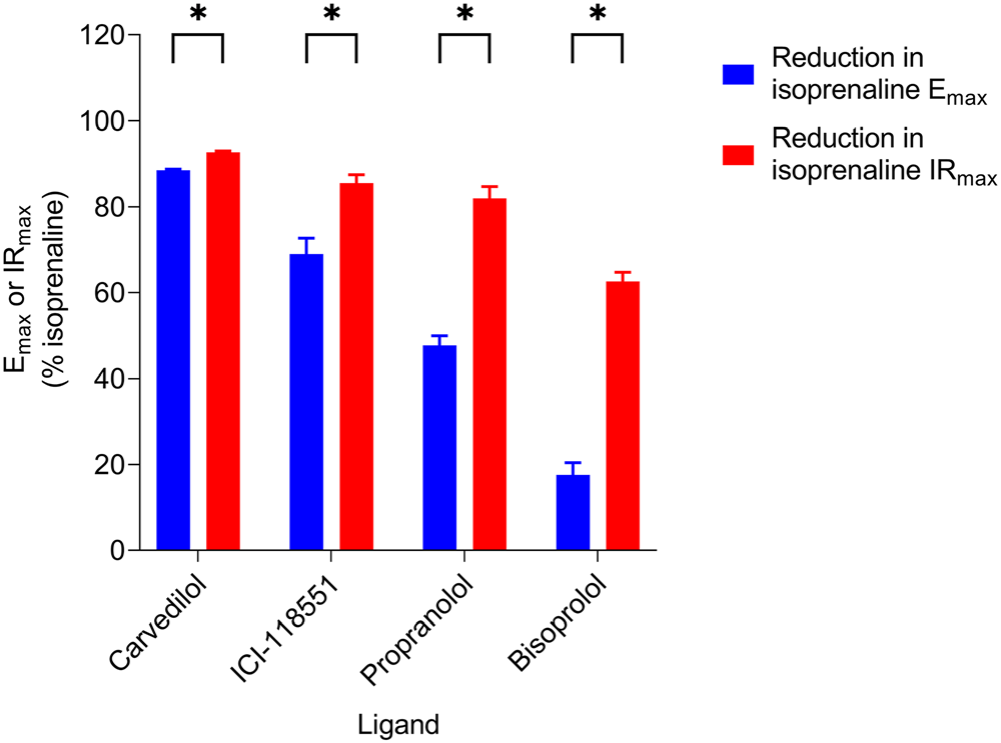
Comparison of the reduction in mean isoprenaline E_max_ and IR_max_ values relative to 1 μM isoprenaline following 30 min preincubation of carvedilol, ICI‐118551, propranolol (all 100 nM) and bisoprolol (10 μM). Data points represent mean ± SEM from five independent experiments (n = 5). Significant differences are indicated, determined by an unpaired *t*‐test. *P* < 0.05 was used as the level for significance (*P* > 0.05 = no significance [ns], *P < 0.05).

A classic feature of low receptor expressing cell systems exhibiting transient agonist‐responses, is the phenomenom of hemi‐equilibria in antagonist competition experiments, where the antagonist does not dissociate sufficiently from the receptor within the time‐scale of the agoinst peak response. In the present study, the effect of 30 min preincubation of increasing concentrations of various competitive β_2_‐adrenoceptor antagonists/inverse agonists (carvedilol, ICI‐118551, propranolol and bisoprolol) on the time‐course of isoprenaline responses was measured. These data show that the addition of the antagonists suppressed both the peak response and the initial rate of signal generation achieved by isoprenaline (Figure 6). Table 3 shows the E_max_, IR_max_, log EC_50_ and log L_50_ values for isoprenaline in the presence and absence of the antagonists. All E_max_ and IR_max_ values are normalised to 1 μM isoprenaline in the absence of antagonist. Except for bisoprolol, application of each of the antagonists caused a large concentration‐dependent reduction in the E_max_ achieved by isoprenaline (Figure 6a–d). In each case, the reduction in the maximal response reached a plateau, whereby further increases in antagonist concentration did not reduce the maxima further but rather shifted agonist log EC_50_ values to higher agonist concentrations. Bisoprolol, however, only produced a significant reduction in the maximal response at the highest antagonist concentration used (10 μM), instead eliciting a parallel rightward shift in the agonist concentration‐response curves at lower antagonist concentrations. The degree of reduction of the E_max_ by the antagonists correlates with the order of their respective dissociation rates at the β_2_‐adrenoceptor according to Sykes et al. (2014), whereby the slower the dissocation rate of the antagonist, the more drastic reduction of the response maxima – carvedilol dissocation rate: 0.033 ± 0.006 min^−1^ < ICI‐118551: 0.21 ± 0.03 min^−1^ < propranolol: 0.46 ± 0.05 min^−1^ < bisoprolol: 6.86 ± 2.09 min^−1^ (Sykes et al., 2014). Similarly, application of the antagonists also caused a concentration‐dependent reduction in the isoprenaline IR_max_ (Figure 6e–h). As illustrated in Figure 7, for each antagonist this effect was larger than the reduction caused to the E_max_ and even bisoprolol caused a large reduction in the maximal initial rate (Table 3). No difference was observed between the log shift in isoprenaline EC_50_ and L_50_ values by any of the antagonists at their maximal concentrations (Table 3).

**TABLE 3:** Isoprenaline E_max_, IR_max_, log EC_50_ and log L_50_ values ± SEM in the presence and absence of increasing concentrations of several β_2_‐adrenoceptor antagonists/inverse agonists from concentration‐response curves obtained by cAMP GloSensor™ in HEK293G cells from five independent experiments (n = 5)

Antagonist	Log [antagonist] (M)	Isoprenaline E_max_ (% 1 μM isoprenaline)	Isoprenaline IR_max_ (% 1 μM isoprenaline)	Log isoprenaline EC_50_ (M)	Log isoprenaline L_50_ (M)
Carvedilol	0	100	100	−8.26 ± 0.04	−8.17 ± 0.03
	−9	36.50 ± 4.82^*^	36.86 ± 6.91^*^	−8.15 ± 0.11	−8.05 ± 0.11
	−8	14.40 ± 0.63^*^	10.13 ± 0.54^*^	−7.04 ± 0.11^*^	−7.17 ± 0.17^*^
	−7	11.46 ± 0.26^*^	7.31 ± 0.34^*^	−5.82 ± 0.08^*^	5.90 ± 0.10^*^
ICI‐118551	0	100	100	−8.16 ± 0.13	−8.22 ± 0.13
	−9	54.84 ± 3.45^*^	57.71 ± 5.91^*^	−7.92 ± 0.09	−7.99 ± 0.07
	−8	37.84 ± 3.45^*^	22.49 ± 1.93^*^	−7.41 ± 0.18^*^	−7.79 ± 0.16
	−7	31.00 ± 3.70^*^	14.49 ± 2.01^*^	−7.05 ± 0.09^*^	−7.47 ± 0.12^*^
Propranolol	0	100	100	−7.83 ± 0.07	−8.00 ± 0.18
	−9	84.74 ± 4.74^*^	63.39 ± 7.28^*^	−7.72 ± 0.11	−7.92 ± 0.07
	−8	53.85 ± 3.36^*^	25.66 ± 2.15^*^	−7.45 ± 0.10^*^	−7.68 ± 0.12
	−7	52.31 ± 2.28^*^	18.06 ± 2.78^*^	−6.55 ± 0.08^*^	−6.80 ± 0.06^*^
Bisoprolol	0	100	100	−7.97 ± 0.13	−8.14 ± 0.05
	−6	92.73 ± 3.85	64.51 ± 2.16^*^	−7.04 ± 0.07^*^	−7.10 ± 0.02^*^
	−5.5	89.37 ± 4.42	48.77 ± 2.55^*^	−6.57 ± 0.06^*^	−6.70 ± 0.06^*^
	−5	82.40 ± 2.85^*^	37.42 ± 2.19^*^	−6.09 ± 0.05^*^	−6.06 ± 0.05^*^

*Note*: Significant differences in isoprenaline E_max_, IR_max_, log EC_50_ and log L_50_ values to those seen in the absence of antagonist are indicated, determined by a one‐way ANOVA with Tukey's multiple comparisons test. *P* < 0.05 was used as the level for significance.

^*^ *P* < 0.05.

## 4 DISCUSSION

The aim of this study was to investigate the kinetics of ligand‐mediated β_2_‐adrenoceptor responses using the cAMP GloSensor™ biosensor in HEK293 cells that express the β_2_‐adrenoceptor at very low endogenous levels (Friedman et al., 2002; Goulding, Kondrashov, et al., 2021, Goulding, Mistry, et al., 2021). The data obtained show that following β_2_‐adrenoceptor agonist stimulation, intracellular cAMP increases rapidly. Regulatory mechanisms such as β_2_‐adrenoceptor desensitisation and breakdown of cAMP by phosphodiesterases then cause the signal to plateau at a ‘peak’ level before ultimately decaying back towards the baseline (Baker et al., 2004; Fan et al., 2008; Moore, Milano, & Benovic, 2007). The ‘peak response' therefore provides a measure of the maximal amplitude of the cAMP response achieved before these regulatory secondary mechanisms become more dominant (Hoare et al., 2020). In contrast, the ‘initial rate' parameter should quantify the initial linear phase of cAMP production following agonist binding before these regulatory mechanisms take hold (Hoare et al., 2020). The maximal initial rate, IR_max_, should therefore provide an indication of the agonist‐occupied receptor's ability to transduce a response prior to regulation, and therefore provide a kinetic measure of agonist efficacy (Hoare et al., 2020, 2022). Consistent with this hypothesis, inclusion of PDE inhibitors in the present study had no significant effect on the initial rates of response but did significantly elevate the peak response obtained (Figure 5).

As expected, both peak and initial rate responses increased with agonist concentration up to a maximum level. The rank order of efficacy of the tested β_2_‐adrenoceptor agonists remained the same in terms of both E_max_ and IR_max_ values (isoprenaline ≥ formoterol > salbutamol > salmeterol). To directly compare the E_max_ values of β_2_‐adrenoceptor ligands with their IR_max_ values obtained from the same datasets, the data were normalised with respect to a maximal isoprenaline response. This showed that the salbutamol and salmeterol IR_max_ values were significantly decreased compared with their E_max_ values, relative to that of the reference ligand isoprenaline, whereas formoterol showed no significant difference. It is well‐established that different ligands are able to stabilise distinct GPCR conformations which may confer varying affinities for binding to intracellular G proteins and β‐arrestins (Rankovic et al., 2016; Shukla et al., 2014). Since receptor coupling to β‐arrestins causes desensitisation by preventing further G protein binding (Moore, Milano, & Benovic, 2007; Shenoy & Lefkowitz, 2011), the rate of receptor desensitisation is dependent on the agonist‐occupied receptor's ability to recruit β‐arrestin. A slow rate of receptor desensitisation would likely cause the rise phase of the time‐course signal to plateau at a slower rate allowing the response to peak at a higher magnitude than under faster desensitisation conditions. This would in turn elevate the measured E_max_, but not IR_max_ (as observed here for salbutamol and salmeterol), which should be independent of this regulation. A similar argument can be made for the relative E_max_ and IR_max_ values obtained with 100 μM forskolin when compared to isoprenaline measured in the same experiments (Figures 2 and 3). Consistent with this, several studies have revealed decreased β_2_‐adrenoceptor desensitisation by salmeterol compared with higher efficacy agonists due to reduced GRK binding, receptor phosphorylation and β‐arrestin affinity of the salmeterol‐bound β_2_‐adrenoceptor (Clark et al., 1996; Gimenez et al., 2015; January et al., 1998; Moore, Millman, et al., 2007; Tran et al., 2004). This has also been shown for salbutamol (also referred to as albuterol) previously (January et al., 1997; Tran et al., 2004).

It is worth emphasising that the measurement of initial rate of signal generation does not account for ligand binding affinity for the receptor (Hoare et al., 2018, 2022). Therefore, at submaximal concentrations of ligands, the ligand association rate may distort observed initial signalling rate values, due to ligand‐receptor binding becoming the rate‐limiting step, rather than the agonist‐occupied receptor's generation of the signal (Hoare et al., 2018, 2022). Salmeterol has a high binding affinity for the β_2_‐adrenoceptor, relative to isoprenaline, formoterol and salbutamol, due to a fast association rate with the receptor but a slow dissociation rate (Sykes et al., 2014; Sykes & Charlton, 2012). However, despite salmeterol's fast association rate with the β_2_‐adrenoceptor, it has been shown to have a slow onset of action (Johnson et al., 1993; Rosethorne et al., 2010). The lipophilic nature of salmeterol likely contributes to this and causes it to partition in the phospholipid membrane (Johnson et al., 1993; Rhodes et al., 1992). This in turn slows the onset of action of salmeterol relative to less lipophilic ligands such as isoprenaline, formoterol and salbutamol which access the receptor directly from the extracellular surface. Salmeterol also has a slower dissociation rate at the β_2_‐adrenoceptor than isoprenaline, formoterol or salbutamol (Nials et al., 1993; Sykes et al., 2014; Sykes & Charlton, 2012), partly due to its high affinity binding to an exosite formed by residues in extracellular loop (ECL) 2, ECL3 and the extracellular ends of transmembrane (TM) 6 and TM7 of the β_2_‐adrenoceptor (Baker et al., 2015; Masureel et al., 2018). This contributes to salmeterol's long duration of action and a longer time to reach equilibrium (Nials et al., 1993; Szczuka et al., 2009). However, these factors do not appear to be key determinants of the reduced IR_max_ values observed here as salbutamol (which shows a similar reduction of IR_max_) is not highly lipophilic (Johnson et al., 1993; Rhodes et al., 1992), has a faster onset of action than salmeterol (Rosethorne et al., 2010) and displays a similar dissociation rate from the β_2_‐adrenoceptor to both isoprenaline and formoterol (Sykes et al., 2014; Sykes & Charlton, 2012). Thus, the reduced rate of receptor desensitisation by the partial agonists is likely to be the key factor in the reduction of IR_max_ values, relative to E_max_.

Preincubation with the slowly dissociating orthosteric antagonists carvedilol, ICI‐118551 and propranolol (Sykes et al., 2014) caused a concentration‐dependent depression of the maximum peak response to isoprenaline. This is consistent with a hemi‐equilibrium where the apparent insurmountable antagonism observed is a consequence of a failure of the competitive antagonist to dissociate sufficiently quickly from the receptor before the peak agonist response has been achieved (Hopkinson et al., 2000). This phenomenon is particularly pertinent to cell systems where endogenous receptor expression is low and there is no receptor reserve to overcome the loss of a proportion of the receptors due to occupancy by a non‐dissociated antagonist (Goulding, Mistry, et al., 2021). In contrast, bisoprolol which has an extremely fast dissociation rate at the β_2_‐adrenoceptor (Sykes et al., 2014), dissociated sufficiently quickly for isoprenaline to reach binding equilibrium (apart from at the highest bisoprolol concentration). The degree of depression of the isoprenaline maximal response (carvedilol > ICI‐118551 > propranolol > bisoprolol) coincided with the relative dissociation rate constants of the four antagonists (Sykes et al., 2014).

The impact of competitive orthosteric antagonists on the maximal initial rate responses (IR_max_) of isoprenaline was more dramatic. Even the rapidly dissociating bisoprolol showed a considerable reduction in the maximal initial rate of response at all antagonist concentrations. This is unsurprising when considering the time of measurement of the initial rate parameter compared with the peak response. The initial rate is determined by fitting the entire time course to take into account the subsequent regulatory mechanisms. However, by definition, it represents the response obtained within the first 0.2–0.5 min after agonist addition. On the other hand, the peak response is generally achieved approximately 2–5 min following addition of agonist (and longer in the presence of PDE inhibitors). This means that at the time of the initial rate measurement, less antagonist‐receptor complexes have dissociated than at the later peak response measurement, further restricting available receptors for the agonist to bind. In this case, even bisoprolol prevents the attainment of equilibrium by isoprenaline in the time‐frame required for the measurement of initial rate. If the residence times of each antagonist are calculated (based on the reciprocal of the dissociation rate constants published by Sykes et al. (2014)), this gives values of 30.3 min (carvedilol), 4.8 min (ICI‐118551), 2.2 min (propranolol) and 0.2 min (bisoprolol), which are entirely compatible with the above observations. This suggests that the impact of antagonists on agonist E_max_ and IR_max_ measured in the same dataset can provide real insights into the kinetic properties of each antagonist as well as their binding affinity when receptor reserves are low.

In summary, analysis of the kinetic profiles of ligand‐mediated β_2_‐adrenoceptor responses using the cAMP GloSensor™ biosensor in HEK293 cells has enabled simple comparisons of E_max_, EC_50_, IR_max_ and L_50_ values from the same dataset for agonists of different efficacies. This analysis has revealed differences in the relative E_max_ and IR_max_ values for the partial agonists salbutamol and salmeterol with respect to isoprenaline which are consistent with their reduced susceptibility to cause receptor desensitisation. Furthermore, comparison of the effect of different orthosteric antagonists on the E_max_ and IR_max_ parameters for isoprenaline has revealed important differences in their dissociation rate profiles and provides useful insights into the nature of their antagonism in cells with a low endogenous expression of the target receptor. Taken together, this study suggests that valuable new information about the distinct pharmacological and kinetic properties of ligands can be revealed from a detailed kinetic analysis of the full time‐course of agonist‐stimulated responses in living cells.

## AUTHOR CONTRIBUTIONS

Conceived the study: Hill and Veprintsev. Participated in research design: Cullum and Hill. Conducted experiments: Cullum. Performed data analysis: Cullum and Hill. Wrote or contributed to the writing of the manuscript: Cullum, Veprintsev, and Hill.

## CONFLICTS OF INTEREST

DBV is founder of Z7 Biotech Ltd, an early stage drug discovery company. All other authors declare no conflicts of interest.

## DECLARATION OF TRANSPARENCY AND SCIENTIFIC RIGOUR

This Declaration acknowledges that this paper adheres to the principles for transparent reporting and scientific rigour of preclinical research as stated in the *BJP* guidelines for *Design and Analysis* , and as recommended by funding agencies, publishers and other organisations engaged with supporting research.

## Data Availability

The data that support the findings of this study are available from the corresponding author upon reasonable request. Some data may not be made available because of privacy or ethical restrictions.
